# Mechanics of the Tricuspid Valve—From Clinical Diagnosis/Treatment, In-Vivo and In-Vitro Investigations, to Patient-Specific Biomechanical Modeling

**DOI:** 10.3390/bioengineering6020047

**Published:** 2019-05-22

**Authors:** Chung-Hao Lee, Devin W. Laurence, Colton J. Ross, Katherine E. Kramer, Anju R. Babu, Emily L. Johnson, Ming-Chen Hsu, Ankush Aggarwal, Arshid Mir, Harold M. Burkhart, Rheal A. Towner, Ryan Baumwart, Yi Wu

**Affiliations:** 1 Biomechanics and Biomaterials Design Laboratory, School of Aerospace and Mechanical Engineering, The University of Oklahoma, Norman, OK 73019, USA; dwlaur@ou.edu (D.W.L.); cjross@ou.edu (C.J.R.); Katherine.E.Kramer-1@ou.edu (K.E.K.); babua@nitrkl.ac.in (A.R.B.); yiwu@ou.edu (Y.W.); 2 Institute for Biomedical Engineering, Science and Technology (IBEST), The University of Oklahoma, Norman, OK 73019, USA; 3 Department of Biotechnology and Medical Engineering, National Institute of Technology Rourkela, Rourkela, Odisha 769008, India; 4 Department of Mechanical Engineering, Iowa State University, Ames, IA 50011, USA; johnsel@iastate.edu; 5 Glasgow Computational Engineering Centre, School of Engineering, University of Glasgow, Scotland G12 8LT, UK; Ankush.Aggarwal@glasgow.ac.uk; 6 Division of Pediatric Cardiology, Department of Pediatrics, The University of Oklahoma Health Sciences Center, Oklahoma City, OK 73104, USA; Arshid-Mir@ouhsc.edu; 7 Division of Cardiothoracic Surgery, Department of Surgery, The University of Oklahoma Health Sciences Center, Oklahoma City, OK 73104, USA; Harold-Burkhart@ouhsc.edu; 8 Advance Magnetic Resonance Center, MS 60, Oklahoma Medical Research Foundation, Oklahoma City, OK 73104, USA; Rheal-Towner@omrf.org; 9 Center for Veterinary Health Sciences, Oklahoma State University, Stillwater, OK 74078, USA; ryan.baumwart@okstate.edu

**Keywords:** the tricuspid valve, functional tricuspid regurgitation, cardiovascular imaging, mechanical characterization, in-vitro experiments, constitutive modeling, geometrical modeling, finite element modeling, isogeometric analysis (IGA), biaxial mechanical characterization, fluid-structure interactions, material anisotropy, sub-valvular components

## Abstract

Proper tricuspid valve (TV) function is essential to unidirectional blood flow through the right side of the heart. Alterations to the tricuspid valvular components, such as the TV annulus, may lead to functional tricuspid regurgitation (FTR), where the valve is unable to prevent undesired backflow of blood from the right ventricle into the right atrium during systole. Various treatment options are currently available for FTR; however, research for the tricuspid heart valve, functional tricuspid regurgitation, and the relevant treatment methodologies are limited due to the pervasive expectation among cardiac surgeons and cardiologists that FTR will naturally regress after repair of left-sided heart valve lesions. Recent studies have focused on (i) understanding the function of the TV and the initiation or progression of FTR using both in-vivo and in-vitro methods, (ii) quantifying the biomechanical properties of the tricuspid valve apparatus as well as its surrounding heart tissue, and (iii) performing computational modeling of the TV to provide new insight into its biomechanical and physiological function. This review paper focuses on these advances and summarizes recent research relevant to the TV within the scope of FTR. Moreover, this review also provides future perspectives and extensions critical to enhancing the current understanding of the functioning and remodeling tricuspid valve in both the healthy and pathophysiological states.

## 1. Introduction

The tricuspid valve (TV) regulates blood flow on the right side of the heart between the right atrium (RA) and right ventricle (RV) throughout cardiac cycles. Specifically, the TV is responsible for allowing the deoxygenated blood to flow from the RA into the RV during diastole and preventing retrograde blood flow from the RV into the RA as the RV contracts during systole. The TV prevents such backflow into the RA through the closure of the three TV leaflets, namely the *anterior leaflet* (TVAL), *posterior leaflet* (TVPL), and the *septal leaflet* (TVSL). These leaflets are attached to the RA through the ring-like valvular annulus and to the papillary muscles located on the RV by the chordae tendineae. The proper function of these sub-valvular components is critical to overall function of the TV. Alterations to the function or anatomy of the TV can result in a diseased condition called tricuspid regurgitation (TR) that reduces the overall efficiency of the RV function. 

TR occurs when the TV leaflets are unable to completely prevent blood backflow into the RA during systole. Tricuspid regurgitation can be classified by etiology into two categories: *primary* (organic) TR and *secondary* or *functional* TR (FTR) [1,2,3]. On the one hand, TR is considered primary when there is some type of structural abnormality or damage to the TV apparatus as the primary cause of the TR [2]. Congenital diseases, such as Ebstein’s anomaly and hypoplastic left heart syndrome (HLHS), and acquired diseases (e.g., tricuspid leaflet flail resulting from chordae rupture) fall into this category. Interested readers may refer to Table 2.2 from Anwar et al. (2018) [2] for a comprehensive list. On the other hand, TR is classified as functional regurgitation when the TV apparatus itself remains *structurally and mechanically intact*, but instead the TR is secondary to a certain alteration in the surrounding heart geometry/component [1,2,4]. Some examples of the causes of FTR include: RV enlargement, TV annulus dilation, or pulmonary hypertension (cf. Table 2.2 from Anwar et al. (2018) [2]).

FTR often progresses from a combination of three interlinked pathologies that typically stem from a pressure overload or a volume overload in the RV (e.g., pulmonary hypertension) [5,6,7]. First, as a direct result of the pressure or volume overload, the RV will remodel and become enlarged beyond its physiological configuration [5,7]. An early study by Come et al. (1985) [8] observed about a 60% increase in the RV diameter in patients with TR. Consequently, the annulus will begin to dilate away from the septum to form a more circular shape as compared to the healthy elliptical shape. The TV annulus will lose its saddle-like geometry to become more flattened [9,10]. These alterations will continue to progress, resulting in papillary muscle displacement, leaflet tethering, a reduced coaptation of the TV leaflets, and the formation or worsening of FTR [4,5,6,9,10].

**Figure 1:**
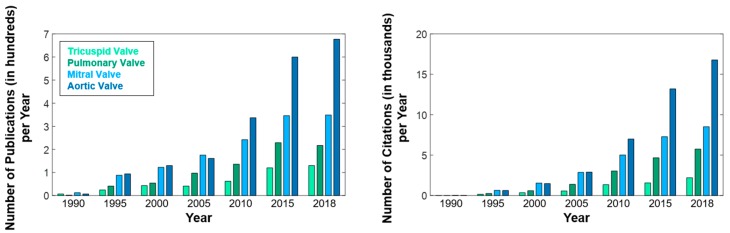
The number of publications (**left**) and the number of total citations (**right**) since 1990 for the four heart valves. Data was adopted from the Web of Science.

TR has been historically ignored in the clinical setting, despite affecting approximately 1.6 million Americans [11,12]. This may originate from the pervasive expectation among cardiac surgeons and cardiologists that correction of the left-sided cardiac lesions will lead to natural regression of FTR [13]. However, recent studies by Dreyfus et al. (2005) and Anyanwu and Adams (2010) [14,15] showed that this over-conservative practice was invalid, and untreated FTR frequently progresses to late severe TR, further worsening long-term prognosis and quality of life. Since the time of those clinical studies, the TV has received increasingly more attention in both the clinical and basic research fields, although less than the mitral valve (MV) and the aortic valve (AV) (Figure 1). Nevertheless, many clinically significant questions still need to be addressed, including the determination of the optimal timing and therapeutic option for treating FTR [14,16,17,18,19,20,21,22], and the understanding of how to mitigate the recurrence of TR after surgical intervention [23,24,25,26,27,28,29,30,31]. As expected, there has been some progress toward partially answering these questions and improving patient-specific therapeutics. For example, recent clinical studies have focused on (i) examining the progression and proper assessment of FTR; (ii) in-vitro and in-vivo studies have been conducted to quantify relevant mechanical properties of the TV and its sub-valvular components; and (iii) computational models have been recently developed to explore TV biomechanical function. Despite these recent efforts to improve the understanding of the functioning TV in both healthy and pathophysiological states, gaps in knowledge still exist in understanding the *underlying mechanisms and recurrence* of FTR. 

In addition to a recent book chapter by Meador et al. (2019) [32], which briefly reviews studies of the biomechanical properties of TV sub-valvular components, this review paper focuses on providing a synopsis and commentary with regard to the recent advances relevant to the TV and FTR. This review paper will also provide an opinion on future perspectives of the TV research for addressing broad topics, such as clinical applications of the presented work and developments critical for patient-specific therapeutics. The remaining sections of this review paper are organized as follows. Section 2 will address the current status of clinical imaging modalities within the scope of the diagnosis and treatment of FTR. Recent advances in understanding the mechanical behaviors and function of the TV using in-vivo and in-vitro methodologies will be discussed in Section 3. In Section 4, current computational modeling tools and related methods will be presented, which aim to enhance the understanding of TV function and disease progression. Concluding remarks, a summary of the key take-away messages, and our perspectives on future TV research will be provided in Section 5.

## 2. Functional Tricuspid Regurgitation: Diagnosis and Treatment Options

### 2.1. Sub-Valvular Structures and Components of the TV

**Figure 2:**
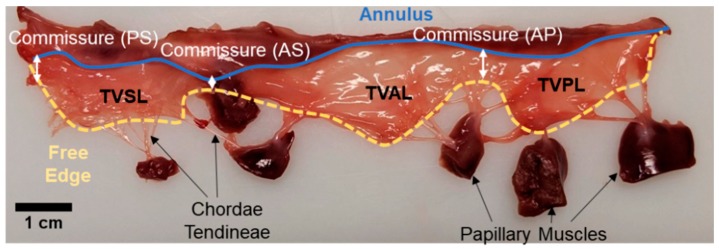
An excised porcine TV tissue sample, showing the three tricuspid valve leaflets, papillary muscle, chordae tendineae, commissures, and the TV annulus.

The TV regulates the flow of blood between the right atrium and the right ventricle of the heart. Each sub-valvular component of the TV (cf. Figure 2) is critical to the organ-level TV function, and the details of each component are discussed as follows.

#### 2.1.1. TV Annulus

The annulus is a fibromuscular ring that encircles the atrioventricular junction, marking the border between the atrial and the ventricular myocardium. The annulus connects the valve leaflets to the heart chambers. Some studies have found the TV annulus to be pear-shaped [33,34], whereas other studies have predominantly found the annulus to be more saddle-shaped [33,35,36]. The configuration of the TV annulus plays a major role in the coaptation, mobility and the stress distribution in the TV leaflets and chordae tendineae [37,38]. The average diameter of the TV annulus at end systole is 3.15 cm [39]. As the annulus curvature increases, the stress in the TV anterior leaflet decreases, and this alteration in the curvature ultimately results in an increased leaflet strain and abnormal tissue remodeling. During disease conditions, the saddle-shaped annulus enlarges, becoming circular, and the corresponding change in the annulus area typically serves as a predictor of valve disorders such as tricuspid regurgitation [40].

Structurally, the annulus forms the base of the TV leaflets and is composed of two types of discontinuous segments—muscular annulus and collagen-rich fibrous annulus (Figure 2) [41]. The muscular annulus is formed of a circumferentially oriented myofiberous lamina and a second lamina formed of myofibers perpendicular to the circumferential myofibers [42]. Racker et al. (1991) [43] described that the anterior, lateral, and posterior regions of the TV annulus are completely encircled with circumferential myofibers with only a thin muscular connection at the medial region of the TV annulus. The fibrous annulus forms the antero-medial regions and continues with the connective tissues into the TV leaflets. Microscope-based study of the human TV annulus [44] indicated the presence of *myofibers* in the posterior and anterior annulus and *collagen bundles* in the septal annulus.

#### 2.1.2. TV Leaflets

**Figure 3:**
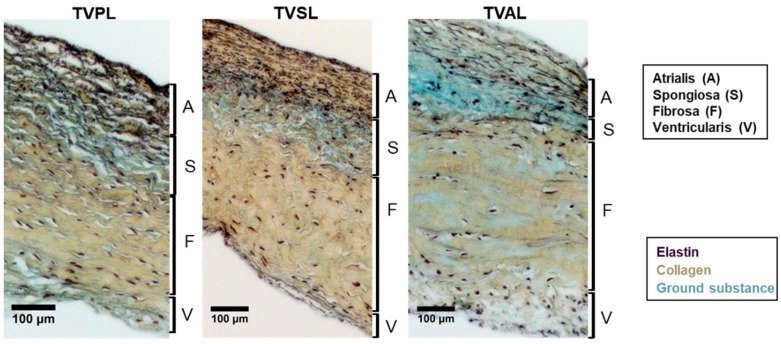
Histological images revealing the porcine TV leaflet microstructure using Movat’s Pentachrome straining to emphasize the elastin, collagen, and non-fibrous ground substance. The four morphologically distinct layers are also illustrated in each image, i.e., A: atrialis, S: spongiosa, F: fibrosa, and V: ventricularis.

The TV annulus transitions into three leaflets: the TVAL, TVPL, and TVSL (Figure 2). In general, the TV leaflets have a rough zone in the crescentic region where chordae tendineae are attached, a broad basal zone at the apex of the leaflet, and a clear zone [45]. Our recent examination of the porcine valves leaflets demonstrated that the TV leaflets are more translucent and thinner than their MV counterparts as a result of fewer collagen proteins [46]. Histological analysis also revealed the difference in the layered structure between the TV and MV leaflets (cf. Figure 10 from Jett et al. [46]). The TV leaflet tissue layers are composed of extracellular matrix proteins—elastin, collagen, proteoglycans (PGs), and glycosaminoglycans (GAGs) populated with dynamic valvular interstitial cells (VICs). The connective tissue structure is organized into four morphologically and biomechanically distinct layers known as the *atrialis* (A), *spongiosa* (S), *fibrosa* (F), and *ventricularis* (V) (Figure 3) [47,48].

A dense, collagenous fiber network distinguishes the main load bearing layer of the leaflets, the fibrosa, from the surrounding tissue. The ventricularis, anatomically situated below the fibrosa and facing the ventricular side of the heart, is rich in circumferentially oriented elastin fibers that assist in the stretching and recoiling of the valve tissue. The spongiosa layer is rich in hydrophilic GAGs and PGs that act as a dampening mechanism during rapid leaflet bending [49,50]. The atrialis layer—on the atrial side of the leaflet—is composed of elastin, collagen and GAGs, and this layer of the TV leaflets is reported to have a high innervation density [51]. In addition, VICs are heterogeneous, dynamic cells distributed throughout the leaflets’ layers [52]. VICs play a major role in maintaining the structural integrity of the leaflet tissues by regulating the extracellular matrix (ECM) scaffold remodeling. Different VIC phenotypes express molecular markers found in myofibroblast and smooth muscle cells (SMCs). The activated VICs produce myofibroblasts and express smooth muscle α-actin as well as other contractile proteins commonly found in the vascular SMCs [53]. It has also been shown that the MV leaflet VICs are stiffer than the cells in the TV leaflets, implying a correlation between the VIC-regulated collagen biosynthesis and transvalvular pressure loading [52].

#### 2.1.3. TV Chordae Tendineae

The chordae tendineae are fibrous strings that originate from the ventricular papillary muscles or from the ventricle wall and transmit tensile force to the leaflets (Figure 2). The chordae split into three segments either soon after their origin or just before their attachment to the leaflets or the commissural region [54]. Chordae tendineae are composed of elastin, GAGs, collagen fibers, and endothelial cells [55]. The chordae tendineae are typically categorized as basal, marginal, strut, or commissural based on their leaflet attachment location [54,56]. Each category is associated with varying length, cross-sectional area, and mechanical properties of the chordae tendineae. For example, the marginal chordae that are connected to the free edges of the leaflets are stiffer than the basal chordae that are attached to the TV annulus [57]. 

### 2.2. Imaging Modalities for Assessing FTR

High-resolution imaging modalities have greatly advanced our understanding of TR and other cardiac abnormalities. Non-invasive imaging techniques used to assess TR include computed tomography (CT), cardiac magnetic resonance imaging (CMRI), and echocardiography. Echocardiography is most frequently employed for diagnosing FTR, but CMRI and CT are increasingly used as a complement [53]. Clinicians use these advanced imaging techniques as a surgical intervention timing-indicator and for preoperative surgery planning.

#### 2.2.1. Echocardiography

Echocardiography, an imaging technique that uses ultrasound waves to image anatomical structures, is the principal modality used to diagnose TR. In the clinic, physicians assess preoperative, intraoperative, and post-operative states of TR generally by two-dimensional echocardiography (2DE). Echocardiography relies on transducer probes to emit “ultra” sound waves at a frequency inaudible to humans (>20,000 Hz) that rebound off inhomogeneities before "echoing" back to the transducer probe. Higher density structures exhibit greater impedance to the propagation of sound.

Echocardiography is relatively inexpensive, widely available, and capable of evaluating the TV both functionally and morphologically. This technique can be performed at a patient’s bedside, so it is popular for imaging hemodynamically unstable patients. However, the operator-dependent interface of echocardiography causes certain restrictions. Individual sonographers must adapt conventional probe positioning for patients possessing anatomical variance, such as obesity or emphysema, further altering the uniformity of the data between imaging sessions and in comparative patient studies. Furthermore, consistent and reliable landmarks on the right side of the heart are not as common compared to the left side of the heart. More recent echocardiogram advancements have resulted in the development of real-time three-dimensional echocardiography (3DE), better contrasting agents, and multimodal imaging.

**Figure 4:**
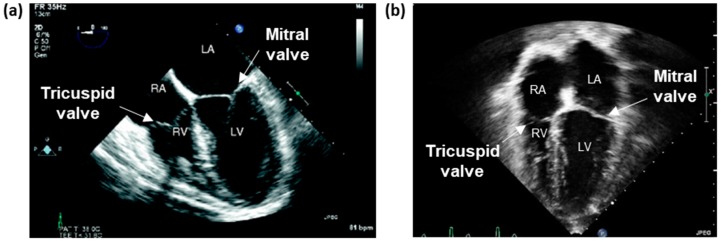
Echocardiographic imaging modalities: (**a**) a four-chamber mid-esophageal view using TEE (image modified from [58]), and (**b**) an apical four-chamber view (A4C) using TTE courtesy of Dr. Mir and Dr. Burkhart from the Children’s Heart Center at the University of Oklahoma Health Sciences Center (OUHSC).

Two major methods exist for performing both 2DE and 3DE: (i) transthoracic echocardiography (TTE) and (ii) transesophageal echocardiography (TEE). For TTE, the transducer probe is positioned noninvasively over the heart. Conversely, in TEE, the probe is inserted down the esophagus to access the heart more directly. While TTE continues to be the cornerstone of diagnostic cardiac ultrasound, TEE offers value as a supplementary tool due to the close probe proximity, decreased signal attenuation, and absence of impedance from intervening lung and bones (Figure 4).

##### Two-Dimensional Imaging Modalities

A 2DE device monitors cardiac image data in B-mode, M-mode, or Doppler. B-mode ultrasonography, or brightness mode, provides 2D grayscale images about a cross-sectional area. Structure brightness can be defined, in decreasing order, as hyperechoic, hypoechoic, and anechoic. High-density structures, such as a calcified valve, reflect most of the sound, resulting in a hyperechoic appearance. In contrast, fluid-filled structures possess low impedance, appearing anechoic. B-mode only provides the most basic image data and, consequently, it is used in conjunction with M-mode or Doppler to convey further information. M-mode ultrasonography, an abbreviation for “motion” mode, uses a rapid succession of pulses along a single ultrasound beam to produce a video-like illustration. By positioning the transducer in a fixed location, B-mode images are recorded at each pulses and changes in the corresponding echo are displayed as a function of time. Valve leaflet coaptation or myocardium movement afford physicians quantifiable time-based data to better interpret the current state of cardiac functionality. Conversely, Doppler ultrasonography depicts the blood velocity, typically showing blood flow toward the device in red and flow away from the transducer in blue. This technique allows physicians to screen patients for cardiac abnormalities, such as TR, by visualizing the regurgitant jet. 

##### Standard Echocardiography Imaging Windows

Due to the complex, multi-component structure of the TV, 2DE requires the acquisition of images from multiple locations to capture the valve’s overall 3D geometry and function comprehensively. The right side of the heart is viewed from the mid-esophageal (ME) (30–40 cm) or transgastric (40–45 cm) windows. The views generally used to image the TV include the right ventricular inflow-outflow ME and four-chamber ME (Figure 4a) (transducer angle: 0–20° and 60–90°, respectively) and the basal short-axis and RV-inflow trangastric views (transducer angle: 0–20° and 100–120°, respectively). Also notable in assessing FTR are the views that delineate the RA and RV. 

**Figure 5:**
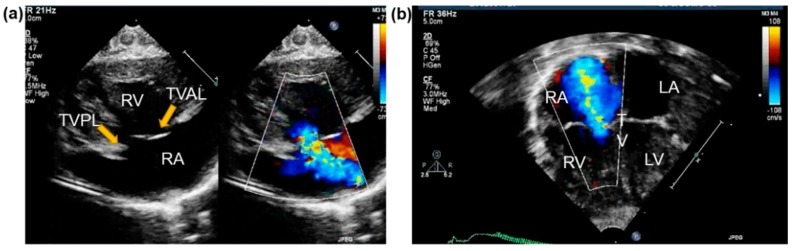
Visualization of severe tricuspid reguritation using two different 2DTTE views and color flow Doppler: (**a**) a parasternal long-axis inflow view, and (**b**) an apical four-chamber view of a newborn with a severe pulmonary hypertension due to diaphragmatic hernia. Images courtesy of Dr. Mir and Dr. Burkhart from the Children’s Heart Center at the University of Oklahoma Health Sciences Center (OUHSC).

The traditional approaches for TTE include right ventricular inflow (RVIF), parasternal short-axis (PSAX), parasternal long-axis (PLAX) (Figure 5a), apical four-chamber (A4C) (Figure 4b and Figure 5b), and more recently, right ventricular-focused (RVF). Addetia et al. (2016) [59] analyzed the efficacy of these traditional views compared to six nonstandard 2D views devised by their group. Using multiplanar reconstruction of three-dimensional data sets, they showed that their novel 2D views accurately identify the TV leaflets based on defined landmarks and anatomical clues. Such nonconventional imaging protocols may be beneficial for further evaluating TV leaflet pathologies.

##### Three-Dimensional Imaging Modalities

Unlike the MV or AV, the complex, nonplanar structure of the TV makes simultaneously capturing the three TV leaflets in one cross-sectional view nearly impossible using only 2DE imaging. Real-time three-dimensional echocardiography (RT3DE) supplements 2DE with detailed anatomical measurements in 90% of patients [60], and allows for concomitant visualization of the opening and coaptation of the three leaflets through the cardiac cycle [61]. Moreover, in a comparison of 2D TTE and RT3DE, Anwar et al. (2007) [62] concluded that RT3DE more reliably assessed the tricuspid valve annulus (TA) size and function. Accordingly, the advent of RT3DE has prompted numerous studies to elucidate the specific valve geometry and anatomy in healthy patients [23,63].

3DE acquires volume data via transducer probes containing a special matrix array of 2500 piezoelectric crystals that can be independently activated, focused, and steered to scan a pyramidal volume of tissue in three dimensions [64]. In addition to the real-time live mode, the 3DE transducer can also obtain full-volume data, which are the merging of information over four consecutive cardiac cycles using a wide angle to cover a larger region of interest. This allows the ability to view both atrioventricular valves simultaneously. However, full-volume imaging possesses limitations in the potential for poor image and spatial resolution due to physiologically-based artifacts. Additionally, the need to suspend respiration for four cardiac cycles during imaging excludes patients with atrial fibrillation or dyspnea.

The three main RT3DE imaging views are *parasternal*, *apical*, and *subcostal*. In selecting one of these views, it is important to consider the response of an imaging system to a point object, known as the point-spread function of the system, which varies in degree according to the system dimensions in use. Standard RT3DE systems employ a dimension of approximately 0.5 mm (axial), 2.5 mm (lateral), and 3 mm (elevation), and thus, the best images (i.e., least distortion or blurring) are acquired in the axial dimension. Conversely, the elevation dimension produces the greatest degree of spreading [61].

#### 2.2.2. CMRI

MRI technology applies a strong magnetic field to align the body’s protons (i.e., hydrogen ions), and radio waves are then generated to disrupt the proton alignment. As the protons realign themselves to the magnetic field, they emit radio signals that the device computer reads and converts into detailed spatiotemporal images [65]. High-resolution, multiplanar images obtained using cine-MRI provide doctors comprehensive information about the morphology and function of the scanned structure. Thus, CMRI may be recommended as a complementary modality to echocardiography in cases of poor image resolution or disqualification from TEE. CMRI is considered the “gold standard” for reliably and accurately measuring the ventricular volumes, ejection fraction, and the myocardial mass [66], which are useful in assessing pre- and post-operative ventricular function in patients with FTR. While MRI produces high-resolution images, the modality is restrictive in practical applications. MRI capabilities are limited in evaluating cases of severe TR due to its inability to be performed on hemodynamically unstable patients. Moreover, patients with permanent pacemakers, implantable cardiac defibrillators, or metal prosthetic heart valves cannot undergo an MRI scan unless they have a newer MRI-compatible system.

#### 2.2.3. Cardiac CT

CT scans use X-ray measurements to create two-dimensional radiographic cross-sections of the heart taken around an axis of rotation. Digital processing yields multiplanar three-dimensional reconstructions of the area of interest with desirable spatial and temporal resolution. Thin image slices allow for detection of distinct valvular boundaries and useful spatial information for assessing RV function [67]. Studies support the prognostic value of cardiac CT for indexing FTR—such parameters include the RA and RV volumes, the leaflet tethering angles and height, and the annular diameter and area [68,69,70]. CT has also been used in the post-operative assessment of annuloplasty ring dislodgment and quantification of the spatial relationship between pacemaker leading in the RV and the associated TR [67,68,71]. Despite its attractive capabilities and applications to the heart valve leaflets, CT exposure must be monitored and limited due to the potential adverse effects of the radiation during the X-ray measurements.

### 2.3. Parameters for Grading TR Severity

Imaging modalities afford qualitative, quantitative, or semi-quantitative analysis of the right side of the heart. Such analyses allow surgeons to index the degree of TR. The most common parameters used to evaluate TR severity include the annular diameter, the size of the backflow jet, the coaptation mode of the leaflets, the width of vena contracta, and the relative size of the TR jet when compared to the RA and dimensions of the right side of the heart, among others. FTR has traditionally been classified into three categories: *mild*, *moderate*, and *severe*, based on the progression of the disease. Presently, the threshold for severe is an effective regurgitant orifice area (EROA) ≥ 40 mm^2^, a regurgitant jet volume (R Vol) ≥ 45 mL, and a vena contracta (VC) width ≥ 7 mm [72]. The establishment of the recommended values for grading TR aid in pre-surgical planning, although, patient-specific variances in the anatomy and pathology, as well as limitations in the current grading scale, confound such recommendations in practice [72].

#### 2.3.1. Regurgitant Jet Area

The degree of TR severity is frequently graded according to the jet area (planimetry of maximal jet area in cm^2^). The regurgitant jet area may be assessed qualitatively or semi-quantitatively. Qualitatively, mild TR displays a small central jet, moderate TR displays an intermediate jet, and severe TR displays a very large central or eccentric wall-impinging jet [73]. Quantitative practices for grading severity of TR are not well-established in contrast to those used for MR. Conventional guides to quantitatively grade TR severity suggest a jet area < 5 cm^2^ to be considered mild, 5–10 cm^2^ to be moderate, and >10 cm^2^ to be severe. However, color flow echocardiography may distort the jet size, prompting inaccurate estimations. Therefore, the European Association for Echocardiography does not recommend the use of the regurgitant jet area to grade TR severity [73].

#### 2.3.2. VC Width

Generally imaged in the A4C view, the VC width (mm) describes the narrowest point of the regurgitant flow before the turbulent jet swells outward, as found immediately distal to the regurgitant orifice. To quantify the VC, the components of the regurgitant jet are identified with pulse wave Doppler (at a Nyquist limit of 50–60 cm/s), and the VC width is measured perpendicular to the jet direction [73]. To obtain a reliable measurement, the VC width is averaged over two to three consecutive heart beats. Recommendations by the European Association for Echocardiography suggest that clinicians rely on the VC measurements when quantifying TR severity: severe TR with a VC width ≥7 mm, while VC width values < 7 mm are considered more difficulty to interpret [73,74]. Major benefits of using VC to grade TR severity include: (i) the measurement is independent of hemodynamic and instrumentation factors; and (ii) it can be used on eccentric jets.

#### 2.3.3. Proximal Isovelocity Surface Area (PISA)

Based on the principles of flow dynamics and continuity (conservation of mass), PISA is another useful parameter for estimating valvular insufficiency [73,75,76]. Aptly named, this measurement attempts to quantify the orifice area through which blood flows. Isovelocity refers to the regurgitant region on the color flow map where the color reverses, and the PISA radius stretches from this point, i.e., the edge of the blue hemisphere, to the center of the valve. The surface area (PISA) is determined using A = 2πr^2^. For assessing TR severity, Lancellotti et al. (2010) [73] recommended a PISA radius ≤ 5 mm as mild, 6–9 mm as moderate, and >9 mm as severe. From the semi-quantitative PISA analysis, quantitative parameters, such as the area of defect, EROA and R Vol, can also be derived [76]. Recommendations for EROA and R Vol are defined only for severe tricuspid regurgitation as ≥40 mm^2^ and ≥45 mL, respectively [72,73].

#### 2.3.4. TA Diameter

Studies have shown that the pathology of the TV varies by patient, and anatomical parameter changes, such as tricuspid annular dilation, may occur in the absence of considerable TR [19]. However, the converse is not true. In a study consisting of 311 patients undergoing MV surgical repair and 148 of who also receiving concomitant TV repair, Dreyfus et al. (2005) [14] determined that FTR does not occur without a pronounced tricuspid annular dilation. Furthermore, the assessment of tricuspid orifices in each of the study’s participants revealed the tricuspid annular dimension to be the only universal feature regarding FTR pathology. Thus, the current threshold for moderate or severe *TR* based on the annular dimension is a diameter > 40 mm; however, this index should be applied conservatively.

#### 2.3.5. Proposed Revisions to The Current TR Severity’s Grading Recommendations

To address the limitations of the current grading system, studies have proposed two additional levels of TR and the inclusion of added assessment parameters, such as the right ventricular early inflow-outflow index. For instance, Go et al. (2018) [72] and Hahn and Zamorano (2017) [77] call for two additional grades of severity, “*massive*” and “*torrential*”, to better describe TR that is already defined as “severe.” The new grading system thresholds “massive” TR as having an EORA of 60–79 mm^2^, an R Vol of 60–74 mL, and a VC width of 14–20 mm. On the other hand, “torrential” TR is defined as having an EROA ≥ 80 mm^2^, an R Vol ≥ 75 mL, and a VC width ≥ 21 mm. 

A recent study [78] retrospectively evaluated the VC width, jet area, EROA, right ventricular early inflow-outflow (RVEIO) index, and the RA and RV volumes using routine TTE data from patients with moderate and severe *TR* (*n* = 395). The RVEIO index was calculated as an integral of the early diastolic filling velocity and the RV outflow velocity during the period of systolic ejection. An RVEIO index ≥ 10, a VC width ≥ 7 cm, a jet area > 10 cm^2^, and an EROA ≥ 0.4 cm^2^ were shown to be independent predictors of TR. The RVEIO index increased incrementally in relation to TR severity, making the RVEIO index another useful parameter for indexing the severity of TR.

### 2.4. Surgical Interventions

Heart valve surgery restores proper leaflet function through one of two methods: (i) surgical valve repair, or (ii) surgical valve replacement. TV repair typically uses one of the following surgical techniques: bicuspidization, classic De Vega, flexible band, or rigid ring. Bicuspidization and De Vega annuloplasty are affordable, simple, and present minimal risk of heart block. However, incidence of residual and recurrent TR is high for bicuspidization and moderate for De Vega annuloplasty. Conversely, ring annuloplasty minimizes residual and recurrent TR but is expensive and more difficult to perform [79]. Valve replacement is generally reserved for patients suffering from comorbidities on the right side of the heart that are unamendable using TV surgical repair [80,81].

#### 2.4.1. Repair Methods for Surgical Treatment of FTR

Because patients classified as having *functional* TR, i.e., regurgitation that occurs due to annular dilation resulting from increased pulmonary or right ventricular pressure, compose 70–80% of cases, most surgeries involve repair of the native geometry [14]. Kay et al. (1965) described the first valve repair technique to treat FTR [82], which involved bicuspidization of the TV (i.e., the complete exclusion of the posterior leaflet) using a suture. More modern suture-based techniques include the De Vega procedure [83], which reduces the annulus diameter while maintaining the tri-leaflet structure, effectively treating the annular dilation at the base of the TV anterior and posterior leaflets that occurs in over 80% of cases [75]. One major limitation of the De Vega suture annuloplasty is that the sutures would tear from the fragile annular tissue resulting in suture dehiscence and recurrent TR. Antunes and Girdwood (1983) [84] proposed a variant of this technique using Teflon pledges to reinforce the annuloplasty suture. De Vega suture annuloplasty is relatively safe and effective for treating minor TR when RV dilation is absent [85]. Both types of suture-based annuloplasty are difficult to reproduce, despite superseding bicuspidization, and the results are unpredictable in comparison to ring annuloplasty [79]. 

**Figure 6:**
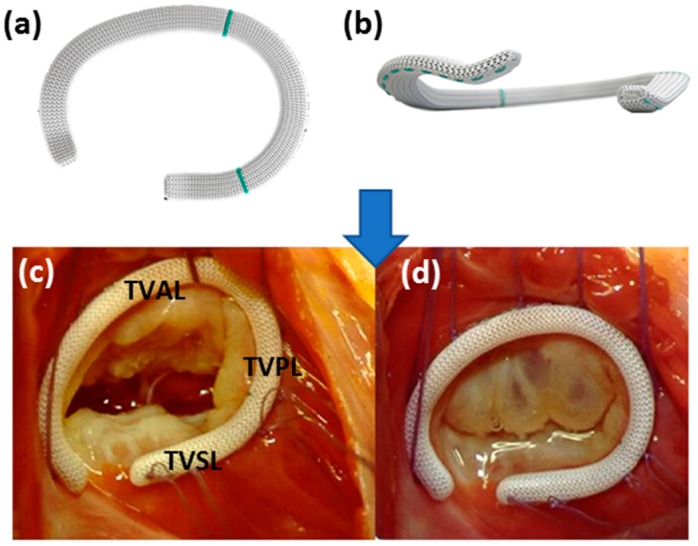
(**a**) Edwards physio-partial annuloplasty ring (**b**) mimics the three-dimensional geometry of the TV annulus. (**c**) The implanted annuloplasty ring is shown during diastole, and (**d**) systole. Images were modified from [92].

Carpentier et al. (1971) [86] proposed the first ring annuloplasty device, a rigid C-shaped prosthetic apparatus, which included a better distribution of tension and a more standardized annular reduction. During surgery, physicians measure the distance between the antero-septal and postero-septal commissures with calipers and select a ring size accordingly. Eight to ten stitches, starting at the midpoint of the septal leaflet and ending at the antero-septal commissure, secure the device to the orifice (Figure 6). Prosthetic rings were adapted from the original idea to include semi-rigid, rigid, and flexible rings. Each type of ring poses advantages and disadvantages. For example, rigid and semi-rigid bands effectively restore and fix the 3D shape of the TV annulus in its native configuration. The most notable advantages of the rigid rings versus the flexible rings are the stabilization and normalization of the septal leaflet dimensions [79]. However, the installation of a rigid annuloplasty ring may increase the forces exerted on the native annulus more as opposed to the forces resulting from a flexible ring. This increase in force may induce annular dehiscence, a well-documented form of valve repair failure [16,30,87,88]. Edwards developed an improved, 3D saddle-shaped, rigid annuloplasty ring to decrease the instances of dehiscence (Figure 6a,b) and improve replicable implantation [89]. The device has demonstrated incredible short-term efficacy, and evaluations are ongoing to assess long-term efficacy. Surgeons must also elect to use partial or complete bands. Partial bands reduce the occurrence of post-operative conduction block by not placing a suture in the region of conduction tissues, whereas complete bands negate the risk of future annular dilation although the septal portion of the annulus is thought not to dilate.

The clinical popularity of both the De Vega suture annuloplasty and the ring annuloplasty has prompted several studies to assess their effectiveness in managing TR during immediate and long-term follow-ups [90]. For example, Rivera et al. (1985) [75] evaluated a randomized study of 159 patients, 76 of whom received Carpentier rings and 83 of whom underwent a modified De Vega procedure. They reported similar results for both techniques in their rates of six-year freedom from 2 + TR. However, the Carpentier ring revealed a more reliable guarantee of long-term freedom from TR (TR recurrence in the De Vega group = 9/19 versus the Carpentier group = 1/16; *p* < 0.01). Another, more recent, study by Huang et al. (2014) [88] compared the treatment outcomes between the suture annuloplasty and the ring annuloplasty. The study observed a significant improvement via post-operative echocardiography assessment of valve function and TR grade (3.4 to 0.6) with no statistical difference between the methods of repair at 1- and 5-year follow-ups (97% and 84% for the De Vega, and 96% and 82% for the ring annuloplasty, respectively). Recurrence-free survival was superior for the ring annuloplasty but not significantly (78.8% vs. 74.5%; *p* < 0.62). More recently, Charfeddine et al. (2017) [91] further documented comparable intermediate survival rates for correcting FTR. In a comparison of ring-based annuloplasty devices, Pfannmüller et al. (2012) [87] demonstrated that patients with rigid bands were at a significantly higher risk of annuloplasty dehiscence. Furthermore, patients suffering from dehiscence exhibited greater residual TR as compared to those without dehiscence. 

Less commonly used techniques for repair include enlargement of the anterior leaflet and “the Clover Technique.” Dreyfus et al. (2008) [93] proposed a procedure to remove the anterior leaflet and replace it with a small tissue section of autologous pericardium. This technique is employed when annuloplasty does not sufficiently correct FTR, such as cases of severe leaflet tethering. On the other hand, “the Clover Technique”, named for the post-operative shape of the valve, was developed by Lapenna et al. (2010) and Belluschi et al. (2018) [94,95] to correct severe FTR and significant leaflet tethering. The procedure uses both suture and ring annuloplasty to restore the annular geometry and a polypropylene suture to fasten each leaflet at the midpoint of the free edge.

#### 2.4.2. Replacement Methods for Surgical Treatment of FTR

Because annular dilation is a consistent pathological feature of FTR, surgical repair of the annular geometry and TV annuloplasty is widely accepted as the preferred method for restoring proper valve function. However, in the event of severe FTR as a secondary pathology, surgeons may replace the diseased valve tissue. Replacement valves may be either mechanical or bioprosthetic. Mechanical valves come in various configurations including disc valves, bi-leaflet valves, and ball valves. Patients with any implanted mechanical valve will depend on anticoagulant medications for the remainder of their lives. This long-term reliance on medicine to viably sustain mechanical valve implants as well as issues with mechanical valve thrombosis led to the biological prostheses as a secondary approach. Bioprosthetic valves may be an autograft (composed of patient’s own tissue), allograft (taken from a donor of the same species), or a xenolog (derived from another species) [96]. Xenograft valves, either porcine or bovine pericardial, are by far the most common valves used in TV replacement. Comparing TV repair and replacement for treating patients with severe TR, no statistical difference was found in early mortality, ten-year overall survival, and ten-year freedom from cardiac death between the patient populations. Thus, TV replacement was concluded to be a viable option for those unsuited for TV repair [81].

## 3. In-Vivo and In-Vitro Investigations

### 3.1. In-Vivo Dynamics and Strains of the TV Annulus

Understanding of the TV annulus has been of high focus in earlier literature, whereas, recently, attention has turned toward understanding the mechanisms of FTR. In TV annulus studies, important measurements include: (i) diameter, measured as an antero-posterior (AP) diameter and a septo-lateral (SL) distance due to the annulus’ elliptical shape, (ii) height, (iii) area, (iv) circumference, and (v) eccentricity, defined as the ratio between the AP and SL diameters. 

**Figure 7:**
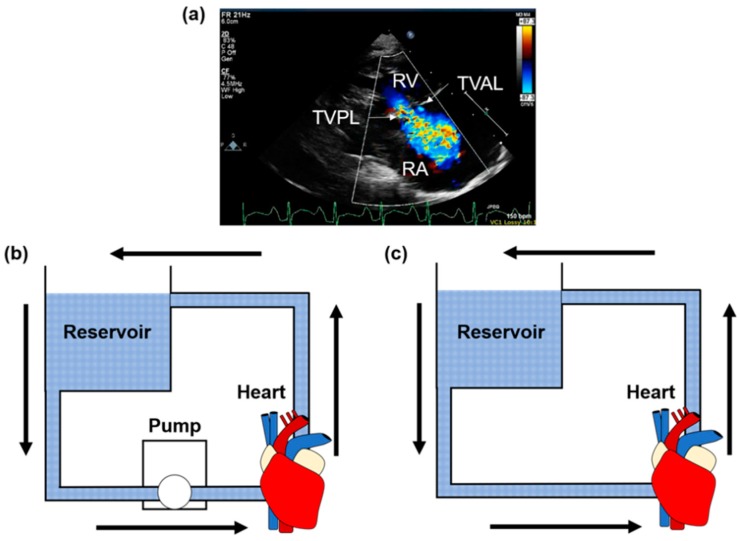
Example methodologies for investigating TV annulus dynamics: (**a**) clinical 3DE in-vivo imaging (image courtesy of Dr. Mir and Dr. Burkhart from the Children’s Heart Center at the University of Oklahoma Health Sciences Center), (**b**) an in-vitro pump-driven fluid flow loop paired with sonomicrometry, or (**c**) a Langendorff model (or a working heart model) paired with sonomicrometry.

In the case of in-vivo studies of the TV annulus, measurements are generally made using MRI, 2DE/3DE, or CT (Figure 7a) [97,98]. A study by Hammarström et al. (1991) [97] used 2DE to measure human annulus parameters with three primary observations: (i) an average annular diameter (between diastole and systole) of 22.5 mm, (ii) the greatest motion occurring along the lateral point of the TV annulus, and (iii) a hinge-point of the annulus movement occurring on the septal side. These observations were later reaffirmed, and more details of the annulus movement were provided through other human in-vivo studies. For example, Ring et al. (2012) [98] used 3DE to quantify: (i) AP and SL average diameters of 41.15 mm and 33.75 mm, respectively, (ii) heights from diastole to systole of 4.2 mm to 5.5 mm, respectively, (iii) areas from diastole to systole of 1145 mm^2^ to 1049 mm^2^, respectively, (iv) perimeters from diastole to systole of 124 mm to 120 mm, respectively, and (v) eccentricity values from diastole to systole of 1.20 to 1.29, respectively. Regarding the annular movement, during diastole the annulus has a more circular shape, while during systole the annulus becomes more elliptical, as shown through the observed eccentricity values. 

The exception to the use of imaging modalities in patients for in-vivo assessment is through open-heart surgery in ovine animal studies in conjunction with sonomicrometry [33,99,100,101]. To briefly elaborate, sonomicrometry uses piezoelectric transducers, or crystals that are fixed to the anesthetized animal’s valve structures by sutures through an open-heart surgery. Once the animal’s heart is restored to its healthy hemodynamic profile, the transducer is then energized using a short electrical pulse. This pulse then generates an acoustic wave that is captured through ultrasound technologies. Sonomicrometry has its limitations, however, in that it can be difficult to ensure that the same crystal locations are used between animal specimen due to intraspecies heart valve geometry variations. Additionally, echocardiography is generally used to ensure a proper crystal placement after surgical operation, although shadowing from the crystals can lead to improper evaluations [102]. 

Rausch et al. (2018) [101] used such techniques to retrieve relevant clinical parameters of the ovine TV annulus, as well as establish engineering metrics (e.g., strain and curvature along the TV annulus). Specifically, they determined that the annular motion is asymmetric in contraction. This asymmetry is elucidated by the deformations originating at the antero-septal and postero-septal vertices, as demonstrated by the contraction concentrations (tangential strain increases up to +0.10 mm/mm), the elongations of the antero-postero vertex, and the mid-septal region of the annulus (relative curvature increase up to +0.03 1/mm). Moreover, Malinowski et al. (2015) [99] used the ovine model to determine the effect of pulmonary hypertension on the TV annular dynamics and found that the disease causes a 12% increase in the total annular area. Employing the same ovine model, Malinowski et al. (2018) [100] also observed the effect of annuloplasty suture on the TV annular mechanics, noting that the tricuspid valve surgical repair resulted in an increase in the compressive TV annular strains. More recently, Mathur et al. (2019) [103] refined the approach by placing four sonomicrometery crystals on each of the three TV leaflets and six on the surrounding TV annulus. Through this approach, they observed that the TVAL and TVPL had similar closing patterns, while the TVSL appeared to have a smaller range of motion and closing velocity. Additionally, their animal study showed that the magnitude of the strain in the belly region of the leaflets was larger than the free edge region and the leaflet strain throughout the cardiac cycle was qualitatively different for both regions. Similar studies have been performed to analyze other pathology-induced changes in the TV annular dynamics [104,105] and the effects of surgical interventions [106].

Although In-vivo studies are useful as they allow for non-invasive, accurate clinical assessment, they are generally limited by the use of imaging techniques that are unable to capture the fine details of valve apparatus movements (usually due to interference with other bodily structures) or fine resolution dynamic information due to limited capture rates. In-vitro studies address this need for higher resolution and dynamic information of the sub-valvular structures of the tricuspid valve.

### 3.2. In-Vitro Flow and Pressure Systems

In-vitro studies are useful for obtaining high-resolution and dynamic information about the annulus movement, usually through a flow system or pressure system (Figure 7b). Flow systems use a pulsatile fluid flow through the valve apparatus to induce TV motions as they would occur in-vivo. However, this system is limited as it can only create passively beating hearts. In the case of passively beating hearts, there is no active ventricle contraction. As such, ventricular pressures applied by an in-vitro flow system cause the inverse annulus action, with expansion occurring rather than contraction during systole. Pressure systems, on the other hand, operate by applying a pressure to the ventricles to force full leaflet closure, allowing analyses of the pressurized valve statically. There is an exception to pressure or flow systems for in-vitro analysis of heart operation to create active beating hearts, known as the Langendorff or the working heart model (Figure 7c) [107]. In these models, the human/ovine/porcine heart is retrieved immediately preceding the human or animal death and the organ is placed into a flow system and perfused with a solution to provide cell nutrients and maintain cell life. The heart is resuscitated and actively beats ex-vivo with the same TV annulus movement that would be found in-vivo. Regardless of the system used for creating ex-vivo beating hearts, the system is paired with a certain imaging modality, such as sonomicrometry, for collecting high-resolution data.

#### 3.2.1. In-Vitro Flow Systems

For studies involving an in-vitro flow system, measurements were usually made using sonomicrometry or 2DE/3DE [100,101,108,109,110]. These studies were generally performed on ovine or porcine hearts because of their availability and lower cost. Imaging analysis of hearts that have been excised from the surrounding bodily structures produces higher-fidelity imaging and information about the TV dynamics compared to non-invasive imaging techniques. For example, a porcine in-vitro study by Khoiy et al. (2018) [108] used a fluid flow system to investigate the effects of chordae rupture on the TV geometry and function. With regard to non-ruptured valves, they found a change in the average diameter for the TV annulus that agrees with the clinical observations for human hearts by Ring et al. (2012) [98]. Focusing on the geometrical changes of the TV with ruptured chordae tendineae, Khoiy et al. (2018) [108] observed that the TV annulus area increased by ~9% during the cardiac cycle.

Moreover, Malinowski et al. (2018) [100] performed a characterization of the TV annular strain using human hearts perfused with donor blood to restore normal beating function ex-vivo (i.e., the Langendorff system). From this study, a mean compressive annular strain of 4% ± 2% was quantified, with the greatest compression occurring at the anterior side and the smallest compression at the septal side. Similar studies have been performed to derive the human tricuspid annulus geometry [111].

In-vitro flow systems could also be useful in measuring the TV leaflet dynamics, as was performed by Khoiy et al. (2016) [109]. Specifically, the porcine TVSL was fixed with sonomicrometry crystals, and the heart was placed in a flow system to emulate human physiologic conditions. The TVSL was shown to have a peak areal strain of 9.8%, a peak circumferential strain of 5.6%, and a peak radial strain of 4.3% at the maximum right ventricular pressure. Furthermore, the areal strain distributions were shown to be *non-uniform* across the TV leaflet, while the principal strains were more spatially uniform. Additionally, the circumferential and radial strains were also shown to exhibit some heterogeneity, with higher circumferential strains at the TVPL and increased radial strains near the TVAL.

#### 3.2.2. In-Vitro Pressure Systems

Pressure systems are primarily useful for analyzing the deformations of the pressurized valve structures during systolic closure. Pant et al. (2017) [112] used a pressure system to examine the change in the alignment of the microstructure of the TV leaflets from a non-pressurized to a pressurized state. For this study, porcine hearts were placed into a system that hydrostatically pressurized the leaflets to force coaptation, and the leaflets were fixed using glutaraldehyde. The primary finding of the study was that a higher alignment of the collagen fibers exists in the pressurized TV leaflets, as opposed to the relaxed, free tissues. Additionally, the leaflet area was found to increase in the pressurized state for the TVAL (405 ± 31 mm^2^ to 479 ± 63 mm^2^) and decrease for both the TVPL (413 ± 23 mm^2^ to 337 ± 26 mm^2^) and the TVSL (429 ± 19 mm^2^ to 374 ± 40 mm^2^). The anisotropy indices of the leaflets were also found to increase by 2 to 3 times in the pressurized leaflets, with the largest increase observed in the TVAL. Similar results have been found in another study by Basu et al. [113].

### 3.3. Chordae Tendineae Force Measurements

Other important dynamic structures of the TV are the chordae tendineae. In a study by Troxler et al. (2012) [114], porcine TVs were excised for use in the Georgia Tech right heart simulator to measure the forces the chordae tendineae experience during the cardiac cycle. In this study, miniature C-ring force transducers were attached to the strut chordae tendineae, and the chordal forces were measured under the normal and emulated pathology conditions. In the normal condition, strut chordal forces ranged from 0.1 to 0.4 N, depending on the papillary muscle and leaflet insertion points. TV annular dilation (100% dilation) caused the chordal forces to nearly double, ranging from 0.2 N to 0.7 N, whereas papillary muscle dysplasia only led to an increase in the chordal force if all three insertion regions were displaced. For combined pathologies, a greater increase in chordal forces was observed when the papillary muscles were moved apically, rather than laterally. 

### 3.4. Biomechanical Quantifications of the Subvalvlar Structures of the TV

With the recent growing interest in the TV’s functions, several in-vitro studies have been performed to analyze the deformations and mechanics of the TV leaflet and chordae tendineae tissues. Some recent studies have assumed the leaflets to be thin membranes and subsequently used Laplace’s Law to estimate the in-vivo mechanical response [46,115,116]. On the other hand, some investigations have determined a parametric spline representation for the leaflets and subsequently used an inverse modeling approach for approximating the leaflets’ mechanical responses [117,118,119,120]. Nevertheless, in-vitro biomechanical analyses using a biaxial mechanical testing technique have been well developed for characterizing soft biological tissues that have distinct transversely isotropic material properties. Within the scope of heart valves, most of the previous literature has been focused on the analysis of the MV or AV leaflet tissues, whereas limited research exists on the TV counterparts (cf. Figure 1). However, as emphasized in Section 1, the TV has become of increasing interest in the past decade since the seminal clinical paper from Dreyfus et al. (2005) [14]. In-vitro studies of the TV leaflets usually focus on biaxial mechanical testing of the tissue under various loading conditions to retrieve the stress-strain responses that may be representative of the physiological deformations of the TV leaflets.

#### 3.4.1. Biaxial and Uniaxial Mechanical Properties of the TV Leaflets

**Figure 8:**
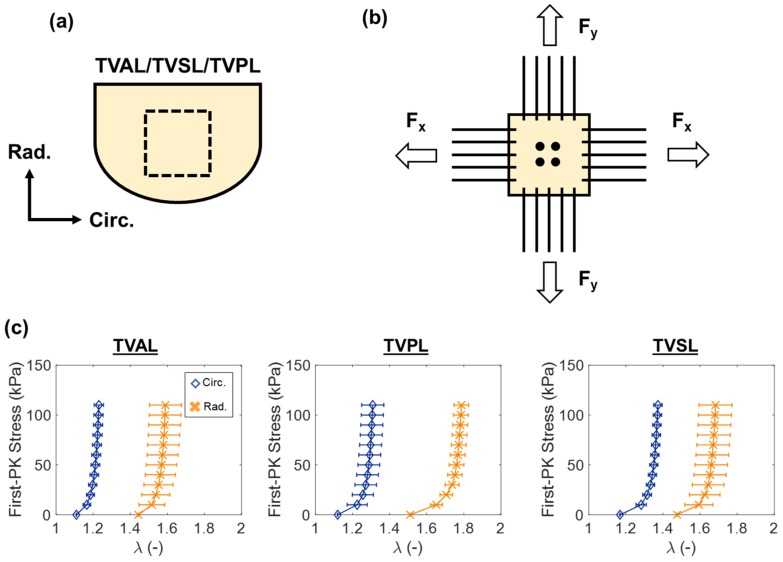
(**a**) Schematic of the excised leaflet tissue and the central bulk region taken from the TVAL, TVPL, or TVSL. (**b**) An illustration of the mounted TV leaflet tissue specimen onto the biaxial testing system with the collagen fiber orientations (circumferential and radial directions) aligned with the testing *x*- and *y*-directions. (**c**) Mean ± SEM of the first-PK stress versus stretch results of the porcine TVAL, TVPL, and TVSL tissues (*n* = 6) under equibiaxial loading protocol (*F*_x_:*F*_y_ = 1:1) at room temperature (22 °C). Images were modified from Jett et al. (2018) [46].

Previous studies have elucidated the leaflet tissue’s mechanical properties by performing biaxial mechanical testing of the central, belly regions of the TV leaflets (Figure 8a,b) [46,116,121,122,123]. Because of the complex microstructure of the fibrous tissues, biaxial mechanical testing is generally performed at various loading ratios of a targeted stress in each tissue direction (i.e., circumferential and radial directions). Coupling between the two tissue directions plays a critical role in the overall stress-stretch responses. Moreover, the TV leaflet tissues exhibit repeatable cyclic mechanical response after the tissue has been preconditioned to restore their in-vivo functionality. 

Several key findings from these biaxial mechanical testing studies of the TV leaflets were summarized as follows. First, the TV leaflets have an anisotropic and non-linear mechanical response, with the radial direction being generally more extensible than the circumferential direction. For example, in Jett et al. (2018) [46], the porcine TVAL tested to a 115 kPa equibiaxial stress was found to have a circumferential peak strain of ~22% and a radial peak strain of ~73%. Second, the mechanics differs between each of the three TV leaflets, with the greatest extensibility and the greatest anisotropy generally observed in the TVPL (Figure 8c). Moreover, Pham et al. (2017) [124] tested *human* TV leaflets to a 70 kPa equibiaxial stress, and they reported circumferential strains of: TVAL, 10%; TVPL, 13%; and TVSL, 10%; as well as radial strains as: TVAL, 17%; TVPL, 22%; and TVSL, 20%. Third, studies have also demonstrated that the TV leaflets’ mechanical responses have a slight dependence on the loading rate. In Jett et al. (2018) [46], the TV leaflets were tested at 2.29 N/m, 4.42 N/m, and 7.92 N/min loading rates, and only a ~7% difference was observed in the tissue’s peak strains. Fourth, the TV leaflets also possess a significant stress–relaxation behavior [46,121,125]. For example, in the study by Laurence et al. (2019) [125], 6 delimited regions of the porcine TVAL were stretched to a 50 N/m equibiaxial membrane tension and allowed to relax for 900 seconds, with a 20–30% decay of the initial membrane tension reported.

In addition, the above-mentioned anisotropic, non-linear nature of the TV leaflet’s mechanical response stems from the gradual recruitment of crimped collagen fibers preferentially oriented in the circumferential direction. Since the crimped collagen fibers do not contribute to the mechanical behavior, the tissue’s mechanical response initially has a long toe-region in which there are large deformations corresponding to low stresses. As the collagen fibers are recruited and straightened, the tissue’s mechanical response stiffens, resulting in the distinct non-linearity. The orientation, recruitment, and re-orientation of the collagen and elastin fibers throughout the TV leaflet tissue under loading has been determined by imaging techniques [126,127,128]. For instance, Alavi et al. (2015) [129] used second harmonic imaging techniques to understand the distribution and orientation of collagen fibers during uniaxial and biaxial loading. Two key findings from their study are: (i) collagen fibers in the superficial layers were aligned in between the radial and circumferential directions when the TV leaflets were in their relaxed state, whereas collagen fibers were oriented in the circumferential direction in deeper layers; (ii) the collagen fibers were reoriented according to the applied biaxial loading.

Another interesting recent study by Basu et al. (2018) [130] analyzed the mechanics of the TV leaflet-annulus transition regions through uniaxial mechanical testing. In their study, they found that the largest Young’s modulus occurred at the septal leaflet-annulus transition (208.7 ± 67.2 kPa), followed by the posterior (136.8 ± 56.9 kPa) and anterior transitions (92.0 ± 66.8 kPa). However, the extensibility of the transition regions was found to be similar across all three leaflet-annulus zones.

#### 3.4.2. Bending Properties of the TV Leaflets

In-vivo, the TV leaflets experience large flexure between the pressure changes of diastole and systole. Biaxial mechanical testing can shed some light on the leaflet mechanical behaviors; however, bending mechanical tests can provide additional insight into the leaflet mechanics. For example, Fu et al. (2018) [131] observed that for the MV anterior leaflet, the leaflet bending angle can be a useful quantitative metric for when clinicians should perform surgical interventions. Thus, it is important to perform such quantifications of the healthy TV leaflets’ bending mechanical behaviors. Bending mechanical testing is typically performed using a custom-made device, based on the device made by Merryman et al. (2006) [132]. This device fixes the leaflet on each end and then allows for a moment to be applied either with or against the natural curvature of the leaflet, and the corresponding displacement is measured using fiduciary markers and digital image correlation techniques. Brazile et al. (2015) [133] used such a device to quantify the bending mechanical behaviors of the TV leaflets. From this study, they observed that when the bending moment was applied with the natural leaflet curvature there was a non-linear momentum-curvature relationship; when the bending moment was applied against the natural leaflet curvature a stiffer behavior was observed. Quantitatively, at a peak change of curvature of 0.075 mm^−1^ the flexural rigidity of the leaflet in the circumferential direction was 54% higher against the curvature than with the curvature, whereas in the radial direction there was a 97% difference. With regard to the instantaneous effective bending modulus similar trends were observed, with the percent difference between the curvatures being 47% in the circumferential direction and 93% in the radial direction. The results of this study can be useful in the development of polymeric replacement leaflets where the materials can be tailored to specific bending mechanical properties [134].

#### 3.4.3. Spatial Variations in Tissue Mechanics of TV Leaflets

**Figure 9:**
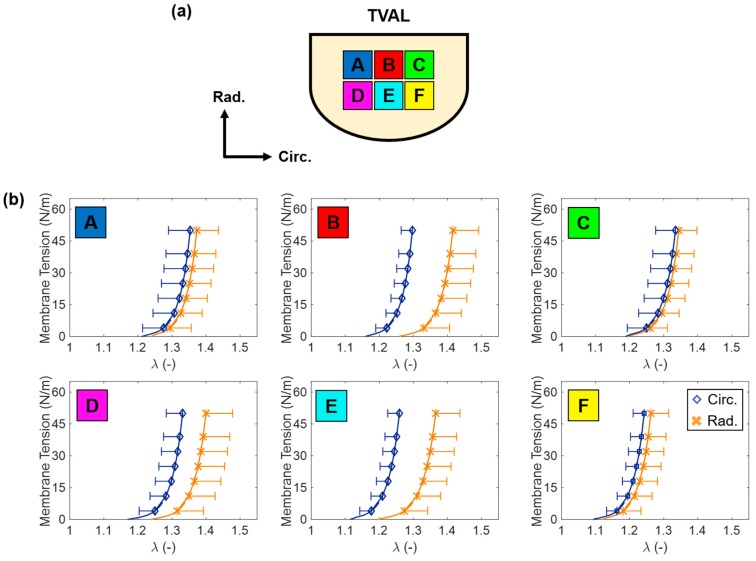
(**a**) Schematic of the TVAL with six smaller tested regions for investigating the regional variance in the tissue’s mechanical properties. (**b**) Mean ± SEM (n = 10~13) of membrane tension versus total tissue stretch results for the 6 tissue regions under the equibiaxial loading protocol. Images were modified from Laurence et al. (2019) [125].

With recent advancements in computational power, models have been refined for the heart valves to consider the leaflets’ complex microstructure, structural heterogeneity, and distinct layers [135,136,137,138,139,140,141,142]. However, these models are typically based on investigations of bulk, central regions of the MV or AV leaflets (cf. Subsection 3.4.1) rather than the spatially varied mechanical properties of the TV leaflets. Our group sought to fill this gap in knowledge by providing a mechanical characterization of the TV leaflet specimens over their spatial domain. In Laurence et al. (2019) [125], we first sectioned the TVAL into six smaller regions (labeled A–F, Figure 9a) that were mechanically tested under our established biaxial mechanical testing protocols [46]. 

This study, which is the first of its kind, demonstrated the spatial variability of the TV leaflet’s mechanical properties (Figure 9b). Specifically, from equibiaxial mechanical testing of the tissues to a 50 N/m membrane tension, central regions of the leaflet (B and E) were observed to exhibit a greater material anisotropy than those edge regions (A, C, D, and F). With regard to the tissue’s extensibility, higher extensibility was observed in regions near the TV annulus (A–C) as compared to regions near the free edge (D–F). In addition, the regions near the TV annulus had circumferential peak stretch values approximately 4% higher than those in the edge regions, whereas a similar, but less pronounced trend was observed for the radial peak stretches (i.e., ~2.5% higher in regions bordering the TV annulus). The results of this study are useful in better understanding the leaflet mechanics and how the stress could vary and be distributed spatially over the TV leaflet.

#### 3.4.4. Microstructural Constituent’s Contributions to Tissue Mechanics of the TV Leaflet

While our study on the regional variance in the TV leaflet tissue’s mechanical properties provides useful insight into the overall biomechanical behaviors of the TV, more detailed biomechanical characterizations could be made to investigate the mechanical contributions associated with the underlying microstructural constituents. For the AV leaflets, the mechanical contributions of each constituent have been comprehensively quantified using a biaxial mechanical testing procedure in which tissues are tested before and after enzymatic removal of the constituent of interest [49,50,143,144]. However, this has not been done for the TV leaflets. Therefore, we sought to fill this knowledge gap by applying this enzymatic-treatment procedure to the TV leaflets [145]. 

**Figure 10:**
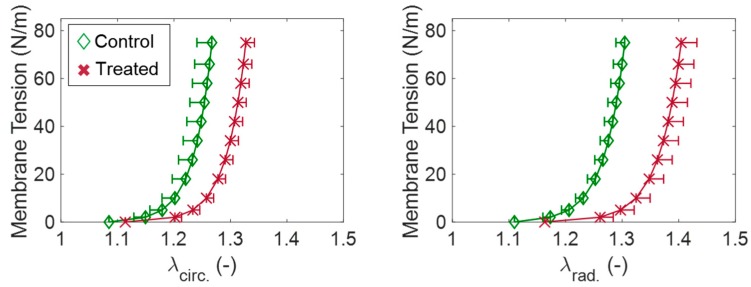
Mean ± SEM (*n* = 6) of the membrane tension versus total tissue stretch results (**left**: circumferential direction, **right**: radial direction) for the TVAL between the control and enzyme-treated groups under equibiaxial loading (*F*_x_:*F*_y_ = 1:1). Figures were modified from Ross et al. (2019) [145].

In our study on the GAG contributions to the TV leaflet tissue’s mechanical properties, we retrieved porcine TVALs and followed a three-step procedure: (1) TVAL tissue specimens were biaxially mechanically tested; (2) enzyme treatment was performed to remove the GAGs; and (3) treated tissues were biaxially mechanically tested using the same procedure as in Step (1). From this study, it was observed that the GAG-removed tissues (treated, T−) experienced greater stretch than those intact (control, C−) tissues (Figure 10). Specifically, in the loading to a 75 N/m equibiaxial membrane tension, the GAG-removed leaflets were 4.7% and 7.6% more extensible in the circumferential and radial directions, respectively, compared to the native leaflet tissues. In addition, stress–relaxation testing at a 75 N/m equibiaxial membrane tension revealed a lesser relaxation behavior of the leaflets after GAG removal treatment in both the circumferential relaxation (C−: 17.1% relaxation, T−: 15.0% relaxation) and the radial relaxation (C−: 16.4% relaxation, T−: 14.5% relaxation). Ongoing investigations are currently being conducted by our group to determine the contributions of *collagen fibers* and *elastin fibers* to the overall biomechanical behaviors of the TV leaflets. This unique study further enhances our understanding of the leaflet microstructural constituents, which can be useful in the fields of tissue-engineered heart valves where the recreation of the microstructure remains an emerging challenge.

#### 3.4.5. Mechanics of TV Chordae Tendineae

The TV chordae tendineae are essential to proper leaflet movement and have been of recent focus in TV literature. In-vivo studies for the CT are useful for obtaining mappings of the chordae distributions and geometries in the valve, which can be helpful for clinical assessments or computational simulations.

**Figure 11:**
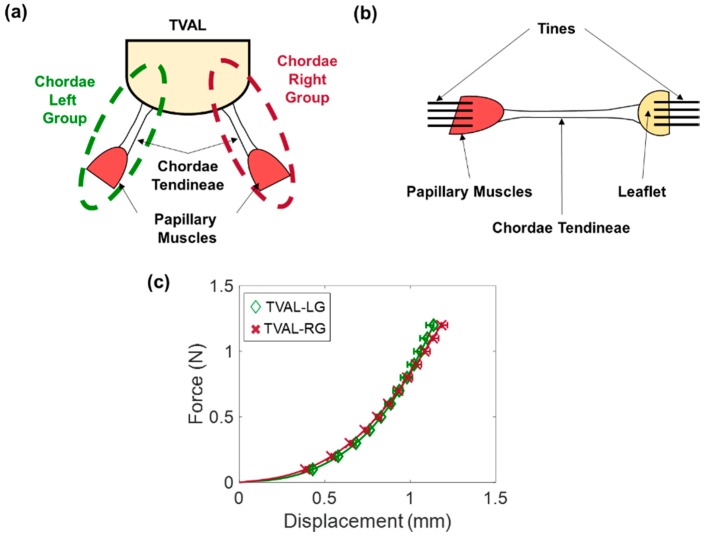
(**a**) The TVAL left (LG) and right (RG) strut chordae tendineae were excised as a group structure, preserving points of attachment to the papillary muscles and leaflet for (**b**) uniaxial mechanical testing to observe (**c**) the mechanical properties of the chordae tendineae as a tissue group.

In-vitro studies of the chordae tendineae are performed under uniaxial mechanical testing, or using strain gauges and a flow/pressure system. Uniaxial mechanical testing is generally performed on chordae tendineae that have been separated from the leaflet and papillary muscles through clamping to a servo-hydraulic tensile testing machine [56,122,146,147,148,149,150,151,152]. Testing protocol then follows either as cyclic loading to a force or stress that is representative of physiologic loading, or as loading until chordal rupture. Our group has expanded on this by excising and mechanically testing a functioning chordae “group” with preservation of papillary muscles and leaflet points of attachment (Figure 11a).

Lim (1980) [146] characterized non-linear mechanical responses for the TV chordae, as well as an increased tissue extensibility with an increased thickness. Comparing the chordae tendineae between the mitral valve and tricuspid valve, the TV chordae had a lower fiber density, resulting in a lower extensibility [146]. More recently, Pokutta-Paskaleva et al. (2018) [122] characterized the mechanical properties of the three porcine chordal subsets (strut, basal, and marginal). In their study, the chordae tendineae were uniaxially loaded until rupture (Figure 11b). Of the three subsets, it was observed that different chordal subsets exhibit different mechanical properties, with basal chords being the most extensible in the TVPL and TVSL (a Green strain of ~15% at the 3 MPa Cauchy stress). In terms of leaflet attachment, the chordae tendineae that attach to the TVAL were observed to have a lower extensibility than their TVPL and TVSL counterparts (TVAL, 0–5%; TVPL, 6–15%; TVSL, 13–16%) [122]. In our group’s study, which analyzed functional strut chordae tendineae groups as opposed to individual chordal segments as done in previous studies, a greater extensibility was observed (Figure 11). At a 1MPa stress, we found that the porcine strut chordae tendineae groups have a peak strain of approximately 11–14%. Our findings (Figure 11c) could provide useful information about the tissue mechanics of the TV chordae tendineae by mimicking the in-vivo functioning environment, which can be implemented in computational models to refine therapeutics related to chordal pathologies, such as chordae rupture. 

## 4. Computational Biomechanical Modeling of the TV

Computational modeling allows for in-silico investigations of TV function that provide unique insight that would be otherwise unobtainable considering conventional in-vitro or clinical methods. The fundamental building blocks of the TV biomechanical modeling framework are selecting the appropriate numerical scheme (e.g., structural modeling, fluid modeling, or fluid-structural modeling), defining the geometry (solid or fluid) and the corresponding computational mesh, specifying the material properties of the tissue and/or fluid domains, prescribing boundary conditions that mimic the clinical scenario of interest, and defining other essential parameters such as contact between leaflet surfaces as well as between blood flow and the TV structure. The selected numerical scheme will use these inputs to provide an approximation of the modeling scenario, such as the closure of the TV. The accuracy of the approximation depends largely on how well the user can describe the complexity of the material, the geometry, and the boundary conditions. Two modeling schemes are typically used in the context of heart valve biomechanics. The first is bio-solid modeling, which describes the behavior of a bio-solid (e.g., heart valves) under specified loading and displacement conditions. The second is fluid-structure interaction (FSI) modeling, which predicts how a fluid and solid will behave and interact through coupled structural dynamics and computational fluid dynamics. Finite element (FE) modeling is typically employed to solve the bio-solid subproblem, while the finite volume method or FE method are both common choices for solving the fluid subproblem. Some research groups elected to use commercially available software packages such as ABAQUS (Dassault Systèmes), LS-DYNA (Livermore Software Technology Corporation), or FEBio (open source) while others opt to develop their own in-house solver. The remainder of this section will emphasize the disparity between the developed computational models for the left-sided and right-sided heart valves, followed by a brief description of recent work for the TV geometrical modeling, constitutive modeling, and computational modeling. 

### 4.1. Disparity of Computational Models for the Left-Sided and Right-Sided Heart Valves

The first 3D FE model for the MV was reported over two decades ago in a study by Kunzelman et al. (1993) [153], whereas the first AV FE model was developed over three decades ago by Hamid et al. (1987) [65]. These early numerical studies contained many assumptions regarding the leaflets’ material and geometrical properties that have since been addressed. For example, Kunzelman’s paper for the MV revealed the anisotropy of the tissue by increasing the Young’s modulus in one direction; in contrast, more recent MV computational models have fully mapped the regionally varying fiber architectures with complex constitutive models to more realistically represent the valve’s function [127,154,155,156,157]. Kunzelman’s early study also used an idealized representation of a porcine MV, while a recent study by Wang and Sun (2013) [158] modeled a patient-specific MV geometry from clinical CT slices. Additionally, FSI models have since been used to obtain more accurate predictions of the MV [159,160], AV [161], or bioprosthetic valve [162,163] dynamics. These advances are closely mirrored with the development of a structural constitutive model [164], patient-specific modeling [165,166], and FSI models [161,167,168]. However, limited research has gone into modeling the TV and pulmonary valve (PV) due to the clinical oversight previously discussed (cf. Section 1). The first study developing a computational model for the TV did not arrive until 2010 in a numerical study by Stevanella et al. (2010) [169] as a direct extension of their earlier MV modeling work [170], which is a stark difference compared to the multiple decades of MV and AV modeling. This delayed development of the TV models has limited the progression toward the refined modeling methods used for the MV and AV, leaving considerable opportunities for future extensions and developments. 

### 4.2. Geometrical Modeling of the TV

#### 4.2.1. Modeling the TV Geometry

One important step toward enhanced TV computational models is accurately modeling the intricate TV geometry. Two common approaches have been used for the TV leaflet geometry: (i) manual or semi-automated segmentation of medical imaging data and (ii) representation of the geometry using cubic splines. Aversa and Carredu (2017) [171] was the first group to perform manual segmentation of the TV geometry for later use in a computational model. Specifically, they performed real-time 3DE imaging of an in-vitro beating heart and then manually segmented the images using a MATLAB framework. Aversa and Carredu (2017) [171] had difficulties accurately capturing the commissures of the TV leaflets, which was likely due to the lack of image clarity in this region. Kong et al. (2018) [172] later manually segmented clinical CT imaging data for three human patients’ TV geometries using the Avizo (Zuse Institute Berlin) software suite. Due to the nature of the in-vivo imaging method, Kong et al. (2018) [172] were unable to directly compare the segmented geometry to the real geometry; however, the three segmented TV geometries were later used in their computational modeling framework and, as discussed in Section 4.4, they obtained excellent closing behavior of the TV. As for semi-automated image segmentation, Pouch et al. (2017) [173] explored the use of semi-automated and multi-atlas methods for modeling the TV geometry for a heart with HLHS, a congenital heart disease. 

The use of cubic splines to represent the complex TV geometry can allow for faster geometry acquisition and the use of the TV geometry within a parametric framework (cf. Section 4.2.2). Stevanella et al. (2010) [169] was the first group to use this method by modeling the TV geometry using cubic splines based on anatomical measurements (e.g., leaflet height, commissure height, etc.) of ex-vivo hearts. Stevanella et al. relied on existing information from literature [33] about the TV annulus shape to create the 3D geometry of the TV apparatus. The later work by Kamensky et al. (2018) [174] also used the spline method, except cubic basis-splines (B-splines) were used rather than cubic splines. Additionally, Kamensky et al. used the annulus and chordae information obtained from the micro-CT imaging data of a formalin-fixed ovine heart to construct a 3D geometry of the TV. We have since expanded on the work by Kamensky et al., (cf. more details in the Section 4.2.2).

#### 4.2.2. Parametric Design of Heart Valve Geometries

Parametric design is typically performed using parameterized computer-aided design (CAD) geometries that are constructed from several selected design variables. Thubrikar et al. (1990) [175] first used a parametric design for heart valve leaflet applications. They considered the AV and introduced a 3D geometry that used a parametric description to search for an optimal prosthetic valve design with improved performance. More recent studies incorporating parametric valve designs include Labrosse et al. (2006) [176], Haj-Ali et al. (2012) [168], Kouhi and Morsi (2013) [177], Fan et al. (2013) [178], Li and Sun (2017) [179], and Xu et al. (2018) [180]. These existing studies have provided guidelines for suitable aortic prosthetic valve designs. However, the overall absence of similar developments in parametric design and modeling of the TV has limited understanding of TV geometry and function.

**Figure 12:**
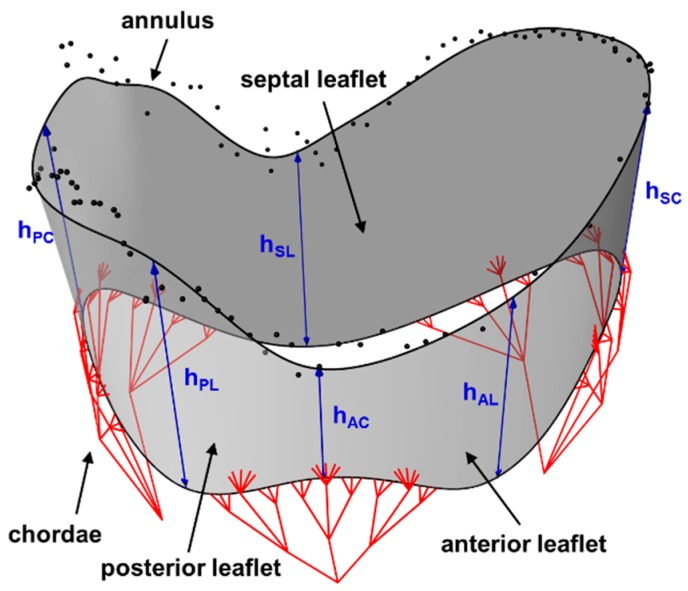
Parametric definition of a B-spline TV surface and chordae. Each leaflet and commissure height is indicated as *h*, where the subscript S, P, or A denotes the septal, posterior, or anterior leaflet, and C or L denotes a commissure or a leaflet location.

**Figure 13:**
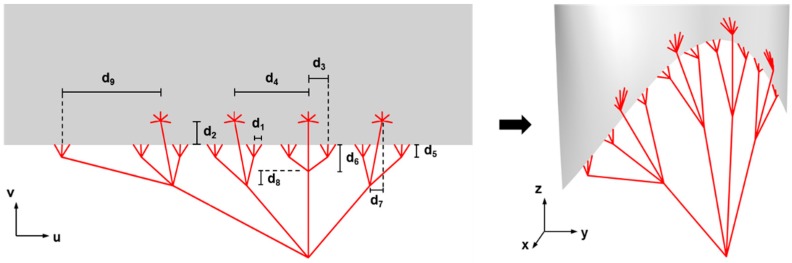
Chordae mapping from the 2D parametric space to the 3D topology.

In this work, we present an extended version of the TV modeling capabilities in Kamensky et al. (2018) [174] that includes a more comprehensive range of valve and chordae configurations. The flexibility of this updated geometry-modeling framework encompasses a variety of possible TV designs, making it effective for many types of TV applications. The highly adaptable framework employs a parametric definition of the valve geometry that enables precise control of the valve. The TV surface and chordae were constructed from a combination of patient data and parameter inputs to obtain the full model of the TV, as shown in Figure 12 and Figure 13. The valve and chordae were then parameterized by the illustrated input parameters, including the leaflet and commissure heights and the distance parameters that control the chordae configuration. The versatility of this framework allows for the modeling of healthy, diseased, and patient-specific TVs while maintaining geometries that are not overly complex. The CAD-based B-spline models produced with this framework can be easily converted into analysis-suitable meshes for FE analysis or directly used for isogeometric analysis (IGA) [181]. The use of these CAD geometries for computational modeling applications will be discussed in Section 4.4.

**Figure 14:**
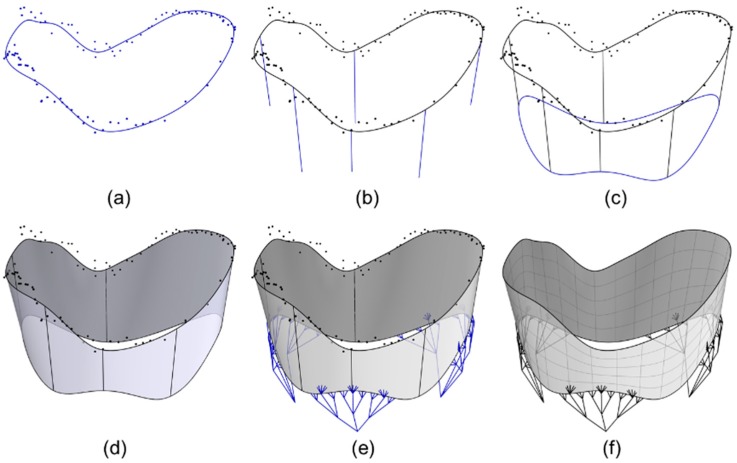
Geometry construction process for the parametric TV: (**a**) B-spline fit of the annulus from the scanned micro-CT data, (**b**) the definitions of the commissure height and the leaflet height, (**c**) a determination of the TV leaflet free edge, (**d**) an interpolation of the TV leaflet surface, (**e**) the attachment of chordae tendineae to the TV leaflets, and (**f**) the final geometry model.

Each step of the geometry construction process within the proposed framework is illustrated in Figure 14. First, the TV annulus scan data from a patient was fit using a B-spline curve that generates the initial annulus shape (Figure 14a). The scan data points that divide the three leaflets were translated onto the fitted annular curve and used to define the location of the commissure and leaflet heights. These input height parameters determined the offset distances of the division points (Figure 14b). The offset points were then interpolated to define the bottom edge of the leaflet geometry (Figure 14c). The offset direction was defined as the normal of the best-fit plane of the annulus data. The set of cubic B-spline curves, two describing the leaflet edges and six defining the leaflet dimensions, generated a bidirectional curve network that was subsequently used to interpolate the valve geometry as a Gordon surface [182] (Figure 14d). The resulting surface can be easily re-parameterized to ensure that the valve surface elements remain suitable for analysis. Finally, the constructed valvular surface was connected to a corresponding set of structured chordae that were parametrically constructed based on patient data (Figure 14e). The chordae distance inputs were set in the parametric space of the B-spline surface, as shown in Figure 13. Using this approach, the attachment locations in the 2D parametric space were naturally mapped into the physical space to determine the actual attachments and spacing of the chordae on the 3D surface (Figure 14f).

**Figure 15:**
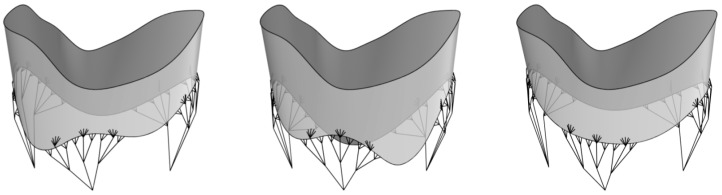
TV geometries with varying leaflet and commissure heights.

**Figure 16:**
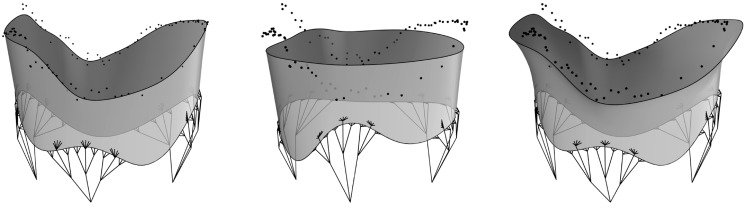
TV geometries of healthy, flattened, and dilated valves.

The versatility of the geometry-modeling framework is further demonstrated in Figure 15 and Figure 16. Specifically, Figure 15 shows examples of varying leaflet geometric parameters, whereas Figure 16 illustrates a comparison of the healthy TV with a flattened and dilated TV annulus. As exhibited, the modeling framework can be flexibly adjusted to accommodate different valve geometry configurations and applications. With this enhanced framework, there are clear prospective developments that would initiate substantial progress in moving TV modeling toward the existing MV and AV modeling capacity. The improved models also have a significant potential to enhance clinical understanding of the TV geometry and provide insight into the function of both healthy and diseased TVs. Besides clinical and surgical applications, the wide range of capabilities offered by this framework also extends the feasibility of developing and analyzing prosthetic valve designs that closely mimic the native TV geometry.

### 4.3. Constitutive Modeling of the TV Leaflets

Despite extensive research in developing constitutive models for the functioning MV, there have been very few similar works for the TV. Much of this gap is primarily due to the limited mechanical characterizations for the TV leaflets until Khoiy et al. (2016) [116] studying porcine TV leaflets, Pham et al. (2017) [124] characterizing human tricuspid leaflets, and subsequently Jett et al. (2018) [46,121] performing extensive biaxial testing on both porcine and ovine atrioventricular heart valve leaflets. Since then, three studies in particular have determined constitutive model parameters for the TV leaflets. However, there is still a significant gap in development of most representative constitutive models for the TV, especially models that are structurally based rather than phenomenological. 

The first study was from Aversa and Careddu (2017) [171]. In this study, the previous human TV leaflet data for equibiaxial mechanical testing from Pham et al. (2017) [124] was used to estimate parameters of an invariant-based strain energy density function *W* with the form [157]:

(1) $$W=C_{10}\left[e^{C_{01}\left(I_{1}-3\right)}-1\right]+\frac{c_{0}}{2}\left[\left(1-\beta \right)e^{c_{1}{\left(I_{1}-3\right)}^{2}}+\beta e^{c_{2}{\left(I_{4}-1\right)}^{2}}-1\right],$$

where *C*_10_, *C*_01_, *c*_0_, *c*_1_, and *c*_2_ are the material model constants, *β* is the parameter describing the material anisotropy (*β* = 0: purely isotropic, and *β* = 1: anisotropic), and *I*_1_ and *I*_4_ are the first and fourth invariants of the right Cauchy-Green tensor **C**. Pham et al. (2017) [124] used this model to fit only the equibiaxial data, which provides an excellent fit as used in their subsequent computational modeling. 

The second study is from Kong et al. (2018) [172] and uses the same human data from Aversa and Careddu (2017) except with a different strain energy density form. In this study, a strain energy density form from Holzapfel et al. (2000) [183] was used, which represents a fiber-reinforced material with two families of fibers (denoted by *i* = 1,2):

(2) $$W=C_{10}\left[e^{C_{01}\left(I_{1}-3\right)}-1\right]+\frac{k_{1}}{2k_{2}}\sum_{i=1}^{2}\left\{e^{k_{2}{\left[\kappa I_{1}+\left(1-3\kappa \right)I_{4i}-1\right]}^{2}}-1\right\},i=1,2.$$

Herein, *C*_10_, *C*_01_, *k*_1_, and *k*_2_ are material properties, *κ* is the model parameter defining the distribution of the family of fibers (κ = 0: well-aligned fibers; κ = 1/3 randomly aligned fibers), and *I_i_* and *I*_4*i*_ are the first and fourth invariants of **C**. Similar to the study by Aversa and Careddu (2017), they only fit the data from the equibiaxial protocol from each TV leaflet, demonstrating a very good fit to the experimental data.

The third study for constitutive modeling of the TV leaflets is from Khoiy et al. (2018) [116]. This extensive study was devoted specifically to constitutive modeling of the TV leaflets, whereas the previous two studies used it in their FE simulations. They used the biaxial mechanical data from their quantifications of porcine TV leaflet mechanical properties [116] to determine the appropriate constitutive parameters for the Fung-type strain energy density form:

(3) $$W=\frac{c}{2}\left(e^{\left(a_{1}E_{CC}^{2}+a_{2}E_{RR}^{2}+2a_{3}E_{CC}E_{RR}\right)}-1\right),$$

where *c*, *a*_1_, *a*_2_, and *a*_3_ are material constants, and *E_CC_* and *E_RR_* are the Green strain in the circumferential and radial directions, respectively. They were able to obtain excellent fits (*R*^2^ > 0.85) for the model considering five different loading protocols (*T*_Circ_:*T*_Rad_ = 1:1, 1:0.75, 0.75:1, 1:0.5, 0.5:1). 

Furthermore, Khoiy et al. (2018) [116] sought to understand how well the material model performed when attempting to predict the TV mechanical behaviors. They fit the Fung-type constitutive model (Equation (3)) to the data of only four of the five loading protocols (*T*_Circ_:*T*_Rad_ = 1:1, 1:0.75, 1:0.5, 0.5:1) and predicted the TV’s stress-strain behavior using the fifth protocol (*T*_Circ_:*T*_Rad_ = 0.75:1). The results from this prediction of the fifth protocol agreed well with the experimental data (R^2^ > 0.88), illustrating the potential for this strain energy density form to be able to fully capture the TV mechanical behavior. In the same study, Khoiy et al. also explored different measures for averaging experimental data prior to performing the constitutive model fitting: the membrane tension, the Cauchy stress, and the first Piola-Kirchhoff stress. They found that membrane tension is sufficient if the tissue thickness does not vary significantly. Otherwise, either the Cauchy stress or first Piola-Kirchhoff (1st-PK) stress must be used. This is currently the most thorough study for determining the constitutive model parameters for the TV leaflets. However, more extensive studies exist for the MV [142,184,185] and AV [141,164] and similar studies are necessary for the TV to move toward a more realistic representation of the TV mechanical response.

In summary, there are limited studies for determining constitutive parameters for the TV leaflets. These studies are not nearly as extensive as those for the MV leaflet tissue where the leaflets have been modeled using detailed structural constitutive models [142,185], or where the effect of selected constitutive model on biomechanical function has been determined using a FE framework [127]. While the phenomenological constitutive model forms used for the TV have shown a reasonable representation of the TV mechanical function, it is essential to not limit TV constitutive modeling to those few forms. Countless other constitutive model forms, both phenomenological and structural, exist in the literature for the other heart valve leaflets, and should be thoroughly examined to determine if they effectively capture the mechanical behavior of the three TV leaflets. These studies should be complemented with investigations to determine the effect of constitutive model form on the biomechanical function of the TV using a patient-specific computational modeling framework.

### 4.4. Computational Models of the TV

Computational models combine the topics discussed in this section to predict the TV biomechanical function. Approaches using bio-solid modeling or the FSI framework have been currently employed for these investigations. Nevertheless, as previously described in Section 4.1, the limited number of computational models is in stark contrast to the numerous present for the MV or AV. While it is evident that more computational modeling investigations are needed, the handful of existing and ongoing studies provide the essential foundation for cutting-edge advancements of TV computational models. For example, the study by Stevanella et al. (2010) [169] provides the fundamental work behind parametric representations of the TV for use in bio-solid models. The work by Kong et al. (2018) [172] is a critical step in modeling patient-specific conditions using a patient’s clinical imaging data. Even more recently, Singh-Gryzbon et al. (2019) [186] have employed FSI modeling of the TV biomechanical function. 

#### 4.4.1. Bio-Solid Models of the TV

The first bio-solid model published for the TV was from Stevanella et al. (2010) [169], in which a parametric representation of the ex-vivo TV geometry (cf., Section 4.2) was used and was subsequently discretized into an FE-suitable mesh. The leaflets were represented by 40,300 three-node triangular plane-stress shell elements and the chordae tendineae were represented by a series of two-node truss elements. To simulate valvular closure, a time-dependent physiological pressure traction was prescribed to the ventricular side of the leaflets while the annulus contracted in accordance with previous values [33]. These simulations were performed using the commercial ABAQUS/Explicit dynamic FE software package. Results from the FE model showed a stress values less than 100 kPa, peak strains of ~52%, and papillary muscle forces ranging from 0.37–0.75 N. It should be noted that these simulations used material properties for the MV leaflets, which may not be the most representative of the TV tissue mechanics. 

The next study for computationally modeling the TV was Aversa and Careddu (2017) [171]. In this study, Aversa and Careddu took a different approach than Stevanella’s study and used 3DE imaging data to inform their bio-solid model geometry (cf., Section 4.2). The FE mesh consisted of three-node triangular plane-stress shell elements for the TV leaflets and a series of truss elements to represent the chordae. For simulating TV closure, a time-dependent pressure traction matching was applied to the ventricular surface, the annulus was prescribed to contract based on their in-vitro measurements, and the papillary muscles were assumed to have no rigid body motion. Results of their FE model showed an incomplete closure of the TV leaflets at peak systole. Furthermore, the stress and strain results were irregular with belly stress values near 300 kPa and peak strains of 0.40 occurring near the chordal attachments, which may be due to the inability to properly define the free margin and commissures of the leaflet, which caused the chordae to not behave incorrectly. 

Kamensky et al. (2018) [174] were the next group to perform bio-solid modeling of the TV. Within this study, Kamensky et al. used B-spline surfaces and curves to represent the TV and chordae geometry within an isogeometric computational framework. To simulate the TV closure, they applied a constant transvalvular pressure of 25 mmHg to the ventricular surface. Using this isogeometric framework, two scenarios were simulated: (i) the healthy TV geometry with no modifications, and (ii) rupturing one of the chordae attached to the TVAL. The results from the chordae rupture scenario showed a significant prolapse of the anterior leaflet into the RA. Although this study is brief in its application, it is the first scenario simulating the TV closure within an isogeometric framework and considering a pathological modification. 

More recently, Kong et al. (2018) reported a more clinically oriented study [172] that aimed to develop a patient-specific FE framework using clinical CT imaging data. The geometries of three patients who were suspected of having coronary artery disease were incorporated into their FE modeling framework, in which eight-node hexahedral elements and two-node truss elements were used for the TV leaflets and the chordae tendineae, respectively. Kong et al. implemented a chordae configuration based on ex-vivo measurements of the chordae tendineae due to the limited spatial and temporal resolutions of the CT imaging modality. For simulating the TV closure, applied a pressure traction of 23.7 mmHg was applied to the ventricular side of the TV leaflets, and dynamic motions of the TV annulus and papillary muscles as obtained from the medical imaging data were prescribed. The results of the FE simulations varied between the three patients’ geometries in comparison to previous simulation studies. The average stress in each TV leaflet ranged from 24–91 kPa, which relatively agrees with the values from Stevanella et al. (2010) [169] but is much lower than the values found in Aversa and Careddu (2017) [171]. Furthermore, the average TV leaflet’s strains (0.12–0.32) we were much lower than previous studies (Stevanella et al. ~0.52; Aversa and Carredu 0.40). 

**Figure 17:**
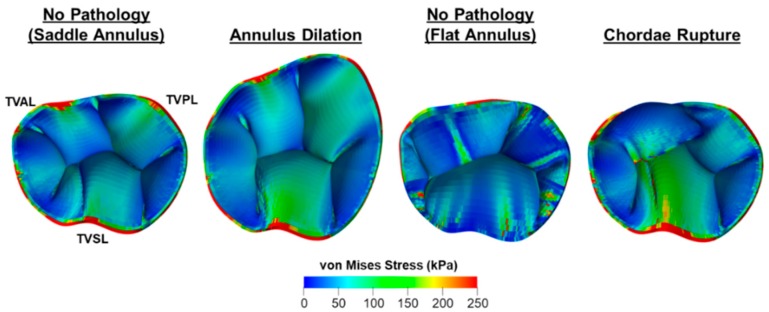
Representative FE results adopted from an ongoing study by Laurence et al. (2019) [187].

In addition, bio-solid modeling has recently been used by our group to better understand the influence of common TV pathologies associated with FTR on the TV mechanical function [187]. Minor modifications to the TV geometry from our previous study [174] were made to investigate five scenarios: (i) healthy (no modifications), (ii) pulmonary hypertension, (iii) annulus dilation, (iv) papillary muscle displacement resulting from left ventricle enlargement, and (v) chordae rupture. Both the mechanics-based quantities, such as the von Mises stress (Figure 17) or Green strain, and the clinically relevant metrics, including the tenting height, tenting area, and the coaptation height, were compared among those study scenarios. Systematic quantifications of these mechanical and/or geometrical changes for each scenario are expected to enhance the current understanding of FTR, especially for situations that may not be possibly replicated under clinical settings.

#### 4.4.2. FSI Model of the TV

Singh-Gryzbon et al. (2019) [186] has recently extended computational modeling of the TV to the consideration of bio-solid/fluid interactions under an FSI framework that would allow evaluations of regurgitant states and other pathological conditions associated with FTR. Specifically, for the bio-solid subproblem, they reconstructed the TV geometry from segmentation of the micro-CT imaging data of an excised, glutaraldehyde-fixed porcine TV at the unloaded (diastolic) and pressure-loaded (systolic) states. The corresponding computational mesh, composed of tetrahedral finite elements (n = 1.20 × 10^5^) with a characteristic length of 0.01–0.02 mm, was then created. Constitutive model forms and parameters previously described by Khoiy et al. (2018) [108] and Toma et al. (2016) [188] were adopted to describe the material properties of the TV leaflets and chordae, respectively. A smooth particle hydrodynamics (SPH) approach [189,190,191,192] rather than the typical FE method under the immersed boundary [193,194,195] or arbitrary Lagrangian-Eulerian [161,196] formulations was employed for modeling the fluid subproblem. This choice was primarily due to the ability of SPH to more accurately capture the separation of flow throughout valve closure and the relatively quick simulation completion time compared to the typical mesh-based methods. The interaction between the bio-solid and fluid subproblems was enforced through a classical penalty-based contact algorithm for the contact between SPH particles and the TV mesh. The boundary conditions considered in their FSI simulations included fixed boundaries for the TV annulus and the base of papillary muscles and a prescribed physiological velocity waveform to the inlet of the fluid domain. Simulations were performed for the healthy scenario with no regurgitation and the regurgitant scenario in which the papillary muscles were displaced to induce FTR. 

When comparing the simulation results from the healthy and regurgitant scenarios, there was an increase in the average leaflet belly stress from 12.45 ± 6.25 kPa to 13.38 ± 7.96 kPa and an increase in the corresponding average principle strain from 0.169 ± 0.099 to 0.1848 ± 0.113. The values presented in this study were markedly lower than previous computational studies that only modeled the bio-solid component of the TV, with the closest comparison being to the study by Kong et al. (2018) [172] (Table 1). Nevertheless, the developed FSI model from this study is a critical first step in moving toward holistic modeling of the dynamic TV biomechanical function.

## 5. Closing Remarks and Future Perspectives

This review paper has summarized recent advances within the scope of assessing or understanding FTR regarding TV biomechanics. First, current image modalities were discussed as well as recent clinical studies that have used the various modalities to gain insight into the development and progression of FTR. Typical therapeutic options for treating patients with FTR were also discussed. Then, recent in-vitro and in-vivo studies involving the TV were surveyed. These studies were primarily focused on understanding the kinematics and/or mechanical behaviors of components of the TV apparatus, including the TV leaflets, annulus, or chordae tendineae. Lastly, several recent TV computational simulation studies were reviewed with key aspects highlighted, such as the constitutive modeling and geometrical modeling. While the existing computational models are broad in the TV biomechanical modeling applications, the stark difference in the number and complexity of computational models for the TV, as compared to the MV/AV counterparts, underscores the emerging need for advances in personalized TV modeling. Such advances would open new avenues for predictive computer simulations to be directly used in clinical settings. 

When understanding the initiation and progression of FTR through in-silico methods, one can look at TV bio-solid modeling as our group has done previously. However, certain aspects of FTR, such as right ventricular enlargement, require interactions between the TV with the RV to completely comprehend the underlying mechanisms. Thus, one future extension within the context of understanding disease initiation and progression is to develop a coupled TV-RV model for fully capturing such essential complex interactions. If developed properly within an FSI modeling framework, a deeper connection between the increase in transvalvular pressure (i.e., pulmonary hypertension) and the subsequent enlargement of the RV or other aspects of FTR could be established. Moreover, this coupled model could be extended to consider both the LVs and RVs with the MV and TVs to explore congenital heart diseases. Some applicational examples include the HLHS where the left ventricle is typically underdeveloped [173,197,198,199], or the Tetralogy of Fallot (ToF) with the four distinct defects affecting flow through the LV and RV [200,201].

Computational models such as those outlined in Section 4.4 or the coupled TV-RV model could also be tailored to clinical applications to provide recommendations concerning proper timing or methodology of clinical intervention. Such clinical extensions would require specific advancements to ensure the timeliness and accuracy of diagnosis or recommendation. For example, a framework would need to be developed that incorporates automated (or semi-automated) segmentation of the patient’s imaged heart geometry, uses this geometry in an inverse bio-solid modeling scheme to quantify the mechanical properties of the TV leaflets, and finally performs in-silico simulations to determine a suggested timing for clinical intervention. These analyses could involve connections made through in-vivo or in-vitro studies, as outlined in Section 3, or through an incorporation of a machine learning model [202,203] trained to recognize key features correlated with necessary or impending clinical intervention. Aspects of this proposed framework could be first tested using an animal model with direct validations using methods such as those described in Section 3 for comparing the mechanical properties of sub-valvular components of the TV.

Certain perspectives must be enhanced or developed for the above-mentioned clinically applicable extensions. First, non-invasive methods for quantifying TV leaflet properties are necessary to capture patient-specific TV mechanical properties. Secondly, one must be able to determine patient-specific boundary conditions derived from clinical imaging data. Lastly, current computational models must be enhanced to realistically model complex FSIs and other bio-solid components of the heart, such as the RV. While these extensions are non-trivial and may take time to properly develop and validate, their collective impacts to the clinical setting will be profound.

## Figures and Tables

**Table 1:** Nomenclature for all the abbreviations adopted in this review paper.

	Abbreviation	Description
Anatomy	AP	Antero-posterior
	AV	Aortic valve
	MV	Mitral valve
	PV	Pulmonary valve
	RA	Right atrium
	RV	Right ventricle
	SL	Septo-lateral
	TA	Tricuspid valve annulus
	TV	Tricuspid valve
	TVAL	Tricuspid valve anterior leaflet
	TVPL	Tricuspid valve posterior leaflet
	TVSL	Tricuspid valve septal leaflet
	VC	Vena contracta
Computational Modeling	CAD	Computer-aided design
	FE	Finite element
	FSI	Fluid-structure interaction
	IGA	Isogeometric analysis
	SPH	Smooth particle hydrodynamics
Disease/Pathology	FTR	Functional tricuspid regurgitation
	HLHS	Hypoplastic left heart syndrome
	ToF	Tetralogy of Fallot
	TR	Tricuspid regurgitation
Imaging and Grading of the TR Severity	2DE	Two-dimensional echocardiography
	3DE	Three-dimensional echocardiography
	A4C	Apical four-chamber view
	CT	Computed tomography
	CMRI	Cardiac magnetic resonance imaging
	EROA	Effective regurgitant orifice area
	ME	Mid-esophageal
	PISA	Proximal isovelocity surface area
	PLAX	Parasternal long axis
	PSAX	Parasternal short axis
	RT3DE	Real-time three-dimensional echocardiography
	RVEIO	Right ventricular early inflow-outflow
	RVF	Right ventricular-focused
	RVIF	Right ventricular inflow
	R Vol	Regurgitant jet volume
	TEE	Transesophageal echocardiography
	TTE	Transthoracic echocardiography
Mechanics	**C**	Right Cauchy-Green tensor
	*E_CC_*	Green strain in the tissue’s circumferential direction
	*E_RR_*	Green strain in the tissue’s radial direction
	*F*_x_	Force in the x-direction
	*F*_y_	Force in the y-direction
	*I*_1_	First invariant of the right Cauchy-Green tensor **C**
	*I*_4_	Fourth invariant of the right Cauchy-Green tensor **C**
	*λ*	Stretch ratio
	*T*_circ_	Membrane tension in the circumferential direction
	*T*_rad_	Membrane tension in the radial direction
Microstructure	A	Atrialis layer
	ECM	Extracellular matrix
	F	Fibrosa layer
	GAGs	Glycosaminoglycans
	PGs	Proteoglycans
	S	Spongiosa layer
	SMC	Smooth muscle cell
	V	Ventricularis layer
	VIC	Valvular interstitial cell
Specimen Labels	-C	Control specimen
	-T	Treated specimen
	A/S	Atrialis/spongiosa layer
	F/V	Fibrosa/ventricularis layer
	LG	Left chordae group
	RG	Right chordae group
